# Evidence of Fine‐Scale Genetic Structure in Tiger Sharks (*Galeocerdo cuvier*) Highlights the Importance of Stratified Sampling Regimes

**DOI:** 10.1111/eva.70117

**Published:** 2025-06-08

**Authors:** Jessica J. Fish, Christine Dudgeon, Adam Barnett, Paul A. Butcher, Bonnie J. Holmes, Charlie Huveneers, Lauren Meyer, Laurent Vigliola, Craig D. H. Sherman, Adam D. Miller

**Affiliations:** ^1^ School of Life and Environmental Sciences Deakin University Warrnambool Victoria Australia; ^2^ School of Science, Technology & Engineering University of the Sunshine Coast Petrie Queensland Australia; ^3^ Biopixel Oceans Foundation Cairns Queensland Australia; ^4^ Marine Data Technology Hub James Cook University Townsville Queensland Australia; ^5^ New South Wales Department of Primary Industries and Regional Development National Marine Science Centre Coffs Harbour New South Wales Australia; ^6^ School of Science, Technology & Engineering University of the Sunshine Coast Sippy Downs Queensland Australia; ^7^ Flinders University, College of Science and Engineering Adelaide South Australia Australia; ^8^ ENTROPIE, SANTECO, Institut de Recherche Pour le Développement Noumea New Caledonia France; ^9^ Cesar Pty Ltd Brunswick, Melbourne Victoria Australia

**Keywords:** eastern Australia, elasmobranch, local genetic structure, ontogeny, population genomics, sex

## Abstract

Understanding the biological connections between populations is essential to wildlife management and conservation. Genetic studies play a central role in characterizing these connections, but typically require stratified sampling regimes to assess the spatial extent and strength of gene flow, and the relative influences of sex and ontogeny on patterns of connectivity. Yet, this can be challenging in some study systems, particularly in large marine species such as sharks, where genetic studies often rely on opportunistic and/or sampling conducted over large spatial scales. We demonstrate the importance of stratified sampling to identify previously undetected genetic structure in tiger sharks ( *Galeocerdo cuvier* ) off eastern Australia, where panmixia has been previously reported. We performed population genomic analyses on 414 tiger sharks, representing males and females and both juvenile‐subadult and adult‐life stages, and 21 locations spanning approximately 3000 km of eastern Australia and the Indo‐Pacific region. Similar to previous studies, we demonstrate a lack of overall genetic structure across the sampling area; however, our analysis shows evidence of spatial autocorrelation and local genetic structuring in juvenile‐subadult female tiger sharks. These results point to potential influences of sex and ontogeny on patterns of population genetic structure and connectivity in Australian tiger sharks. We discuss these findings in the context of essential habitats supporting tiger shark populations and risks of overstating the strength of biological connections among shark populations in the absence of appropriate sampling regimes.

## 1 Introduction

Understanding patterns of connectivity across species' ranges is fundamental to informing the management of biodiversity (Hirschfeld et al. 2021; Hohenlohe et al. 2021). Specifically, understanding the spatial boundaries of populations is critical fort estimating local population sizes and demographic structure (Clark et al. 2024), identifying key habitats for protection (Carr et al. 2017), and predicting the potential spatial reach of disturbance events (Whiterod et al. 2016). This information is also critical to assess the resilience of species to environmental changes, as the mixing of genotypes across environmental gradients can influence the ability of natural populations to adapt to new environmental challenges (Hoffmann and Sgrò 2011; Miller et al. 2020). Genetic studies play a central role in characterising spatial patterns of biological connectivity between populations through estimates of gene flow and genetic structure. However, estimates of gene flow and genetic structure among populations can be misleading in the absence of appropriate spatial sampling or when pooling animals of different sex and life stages for analytical purposes (Phillips et al. 2021). To avoid this issue, stratified sampling regimes, involving replicated sampling at different spatial scales and across sexes and life stages, are often needed to test for evidence of genetic structure at both local and broad spatial scales, and to evaluate the influence of sex and ontogeny on connectivity dynamics (Waples 1998; Holmes et al. 2017; Phillips et al. 2021). Consequently, studies based on opportunistic sampling regimes risk overstating the strength of connectivity across species ranges.

Sampling highly mobile and widely distributed species in a spatially (i.e., transect sampling) and biologically (i.e., across sexes and life stages) replicated fashion for population genetic studies is inherently challenging. For example, large pelagic shark species are often highly dispersive, frequently moving between coastal, neritic, and oceanic habitats, and are difficult to sample due to their mobile, elusive, and often solitary nature (Hirschfeld et al. 2021; Phillips et al. 2021). Population genetic studies have been conducted on approximately 140 shark species to date, many of which have indicated high levels of gene flow and a lack of genetic structure over broad spatial scales both within and between ocean basins (Table 1; Hirschfeld et al. 2021; Phillips et al. 2021). However, most genetic studies on sharks, particularly large pelagic species, have relied on opportunistic and/or sampling conducted over large spatial scales (Table 1). Sampling regimes of this nature often lack the sensitivity to examine the possibility of spatial autocorrelation and local genetic structure (Whiterod et al. 2016; Schmidt‐Roach et al. 2021; Bertram et al. 2022), and to account for differences in dispersal between sexes and different life stages (Phillips et al. 2021). Consequently, it is possible that the connections between populations have been overstated in some shark species due to inappropriate sampling regimes, and that differences in dispersal and genetic structure between sexes and different life stages have been overlooked. Such information is key to informing management aimed at identifying and protecting essential habitats (e.g., nursery or refuge habitats; Barnett et al. 2019; Heupel et al. 2019; De Wysiecki et al. 2023) and understanding important biological factors that underpin the viability of local populations, such as reproductive philopatry, which has been demonstrated across many shark species (Klein et al. 2019; Mourier and Planes 2013; Tillett et al. 2012).

**TABLE 1:** Summary of findings from a selection of population genetics studies on large, highly mobile, and unrelated shark species over the last decade, highlighting sampling limitations.

Species	Ocean basin	Structure within/between ocean basins	Tests for local genetic structure	Tests for sex‐biased dispersal	Tests for ontogenetic differences	References
Tiger shark ( *Galeocerdo cuvier* )	Indo‐Pacific, Atlantic	Within = No; Between = Yes/No^a^	No	No	No	Holmes et al. (2017), Pirog, Jaquemet, et al. (2019), Sort et al. (2021), Bernard et al. (2021)
Broadnose sevengill ( *Notorynchus cepedianus* )	South Atlantic, Oceania, east Pacific	Within = No; Between = Yes	No	No	No	Schmidt‐Roach et al. (2021)
Bronze whaler ( *Carcharhinus brachyurus* )	Indo‐Pacific	Within = No; Between = Yes	No	Yes	No	Junge et al. (2019)
Bull shark ( *Carcharhinus leucas* )	West Indian, Pacific, Atlantic	Within = No; Between = Yes/No^a^	No	Yes	No	Pirog, Ravigné, et al. (2019)
Oceanic whitetip ( *Carcharhinus longimanus* )	Indian, Atlantic	Within = No; Between = No	No	No	No	Sreelekshmi et al. (2020)
Whale shark ( *Rhincodon typus* )	Indo‐Pacific, north Atlantic	Within = Yes; Between = Yes	No	No	No	Vignaud et al. (2014)
Bigeye thresher shark ( *Alopias superciliosus* )	Atlantic, Indian	Within = No; Between = No	No	No	No	Morales et al. (2018)
Shortfin mako (*Isrus oxyrinchus*)	Indo‐Pacific, north Atlantic	Within = No; Between = No	No	Yes	No	Corrigan et al. (2018)
White shark ( *Carcharodon carcharias* )	Indo‐Pacific	Within = Yes; Between = Yes	No	No	No	Blower et al. (2012)

^a^ Holmes et al. (2017) provided evidence of significant genetic structure between tiger shark populations from the Atlantic and Indo‐Pacific Ocean basins, but not between sharks from the Indian or Pacific Ocean basins. Pirog, Jaquemet, et al. (2019) detected weak genetic differentiation between the Western Indian Ocean and Western Pacific Ocean. Bernard et al. (2021) and Sort et al. (2021) discovered genetic differentiation between the Atlantic and Indo‐Pacific. Pirog, Ravigné, et al. (2019) discovered genetic differentiation between bull sharks from the West Atlantic and those from the West Pacific and Indian Oceans, but not within the West Indian and West Pacific Ocean basins.

In this study, we investigate the effect of sampling regime on patterns of genetic structure in tiger sharks ( *Galeocerdo cuvier* ). The tiger shark is a marine top predator with a circumglobal distribution encompassing both temperate and tropical oceanic waters (Simpfendorfer et al. 2001; Dicken et al. 2017). Several studies have indicated significant population genetic structure between tiger shark populations from the Atlantic and Indo‐Pacific Ocean basins, but a lack of overall genetic structure and potential panmixia over vast spatial scales within these basins (Holmes et al. 2017; Pirog, Jaquemet, et al. 2019; Bernard et al. 2021; Sort et al. 2021). However, tiger shark genetic studies have been largely limited to nonstratified sampling approaches (Carmo et al. 2019; Pirog, Jaquemet, et al. 2019; Bernard et al. 2021). To some extent, telemetry studies support the notion of panmixia within ocean basins due to evidence of long‐distance dispersal in some individual tiger sharks (Lea et al. 2015; Lipscombe et al. 2020). However, high variability in movement patterns, both within and between life stages, occurs among tiger sharks tagged in the same locations (Meyer et al. 2009; Fitzpatrick et al. 2012; Holmes et al. 2014; Werry et al. 2014; Ajemian et al. 2020; Barnett et al. 2022; Niella et al. 2022). Consequently, it is possible that a limited number of effective migrants per generation are responsible for driving patterns of genetic homogeneity over broad spatial scales within ocean basins, and that the strength of biological connectivity among tiger shark populations has been overstated. Recently, McClain et al. (2022) provided support for this argument by demonstrating significant genetic structuring among juvenile‐subadult tiger sharks within the north‐western Atlantic Ocean, but a lack of genetic structure among the adult population. These findings are consistent with telemetry studies from the region, suggesting large subadult and adult sharks to be highly mobile, and juveniles and small subadults to show patterns of residency (Sulikowski et al. 2016; Ajemian et al. 2020). However, due to sampling limitations McClain et al. (2022) were unable to test for sex‐biased dispersal or to quantify the spatial extent of gene flow, highlighting the need for more comprehensive sampling approaches.

Here, we build on the findings of McClain et al. (2022) by reassessing the population genetic status of tiger sharks from eastern Australia and the Indo‐Pacific region, where previous studies have indicated a lack of genetic structure across the region based on broad geographical sampling regimes and the use of both modern (i.e., single nucleotide polymorphisms, SNPs) and traditional genetic markers (i.e., microsatellites) (Bernard et al. 2016; Holmes et al. 2017). Tissue samples from more than 400 sharks were collected across 21 sampling locations along eastern Australia and the Indo‐Pacific region. We contrast patterns of genetic structure among sexes and life stages using a spatially replicated sampling design (spanning an ~3000 km latitudinal gradient) and SNP markers derived from reduced genomic representation sequencing. Our results highlight the importance of hierarchical and replicated sampling regimes, providing new insights into the strength of gene flow and connectivity among tiger sharks in the region. Specifically, our results indicate dispersal biases relating to both sex and possibly ontogeny, and the potential for residency and local genetic structuring. These findings challenge the notion of panmixia in tiger sharks from the Indo‐Pacific region and highlight the risk of overstating the strength of connectivity among shark populations in the absence of appropriate sampling regimes.

## 2 Methods

### 2.1 Sampling

**FIGURE 1:**
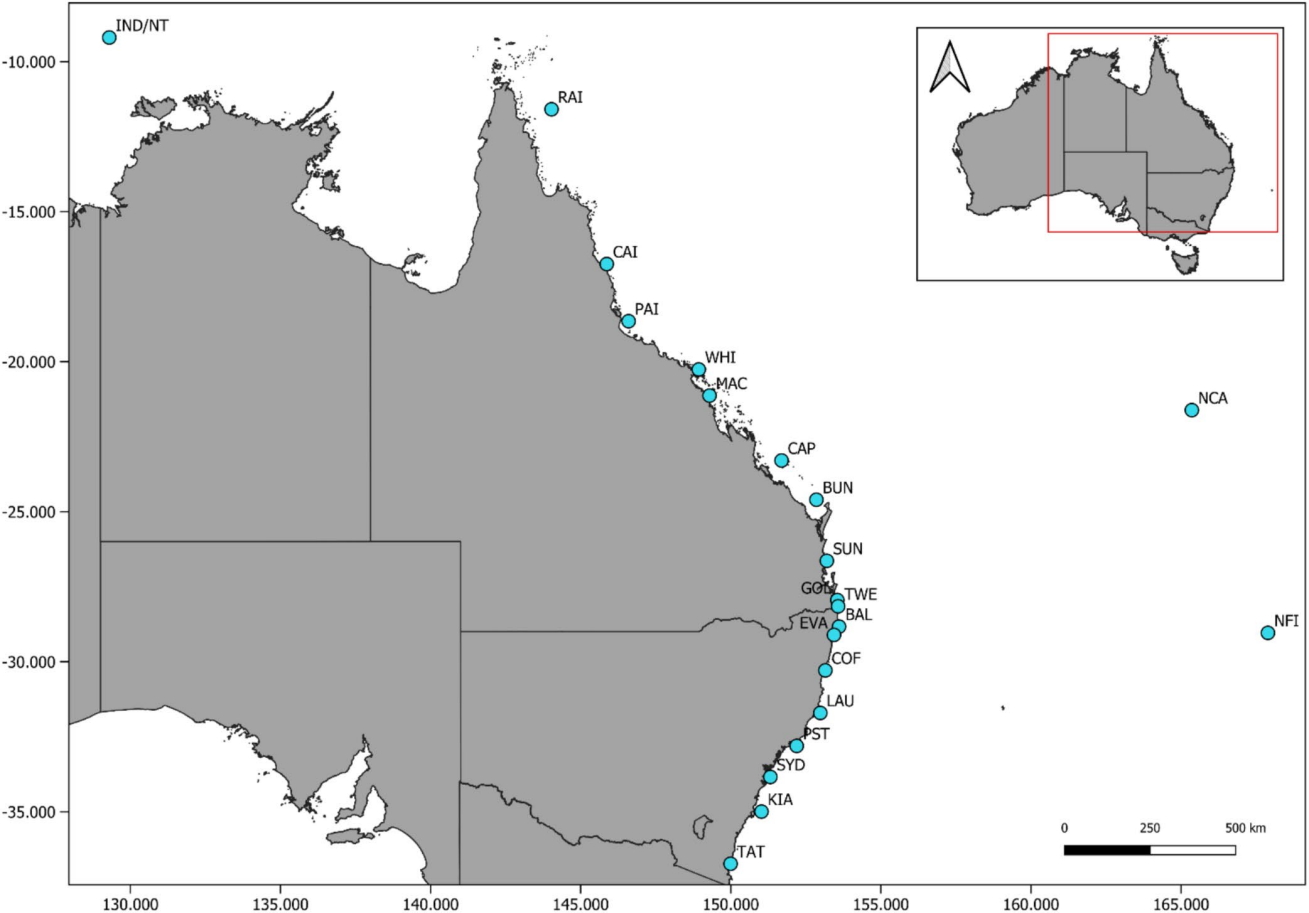
Map of tiger shark sampling locations labeled with site codes (see Table 1) along the eastern seaboard of Australia and the Indo‐Pacific.

Tissue samples were collected from 414 tiger sharks between 2015 and 2021, representing individuals of varying sexes, size classes, and 21 different locations (minimum of five samples per location) spanning more than 3000 km of eastern Australia from Merimbula (−36.9° S, 149.9° E) in southern New South Wales to Raine Island (−11.6° S, 144° E) in North Queensland, and Norfolk Island (−29° S, 167.9° E), as well as New Caledonia (−21.6° S, 165.4° E), and Indonesia/Northern Territory (−9.2° S, 129.3° E; Figure 1). Sharks were captured using hook and line methods (Lipscombe et al. 2020; Tate et al. 2021), or on shark mitigation drumlines and/or nets (Holmes et al. 2017). Live sharks were physically tagged, and all metadata recorded included the location of capture, sex, and body measurements. Muscle and/or fin clip tissue was collected from each shark, immediately preserved in ethanol, and stored at −20°C until required for genetic analysis. The spatially replicated sampling approach used here was designed for the purpose of testing for evidence of broadscale genetic structure as well as local genetic structure and spatial autocorrelation. Replicated spatial sampling of males and females, and both adult and juvenile‐subadult life stages, was also included to allow us to test for sex and ontogenetic biases of dispersal and genetic structure across the sampling distribution (Table 2; Table S1). However, our sampling was biased toward juvenile‐subadult sharks and may be due to sample collection occurring predominantly in coastal nearshore habitats and gear selectivity (Holmes et al. 2012).

**TABLE 2:** Number of individual tiger sharks genotyped at each sampling location, for each sex and life stage (Females > 330 cm TL = adult, Females < 330 cm TL = juvenile‐subadult, Males > 300 cm TL = adult, Males < 300 cm = juvenile‐subadult).

Site name	Site code	Latitude	Longitude	Total *n*	Juvenile‐subadult		Adult	
					Females	Males	Females	Males
Raine Island	RAI	−11.58837	144.03137	8	4	0	4	0
Cairns	CAI	−16.74665	145.87043	18	7	3	8	0
Palm Islands	PAI	−18.65251	146.59898	12	4	2	4	2
Whitsundays^a^	WHI	−20.26174	148.93661	48	7	5	28	8
Mackay	MAC	−21.13224	149.29475	16	11	4	1	0
Capricorn Group	CAP	−23.29548	151.69477	6	3	0	3	0
Bundaberg	BUN	−24.60315	152.85571	29	8	12	8	1
Sunshine Coast	SUN	−26.64017	153.20399	23	16	2	5	0
Gold Coast	GOL	−27.94874	153.55313	6	2	3	1	0
Tweed Heads	TWE	−28.15654	153.5787	10	4	5	1	0
Ballina	BAL	−28.8329	153.6102	77	28	30	14	5
Evans Head	EVA	−29.1104	153.4412	25	12	10	3	0
Coffs Harbour	COF	−30.2922	153.154	28	22	5	0	1
Laurieton	LAU	−31.71084	152.98571	6	4	1	1	0
Port Stephens	PST	−32.80953	152.19961	27	4	5	9	9
Sydney	SYD	−33.8438	151.322	10	2	2	1	5
Kiama	KIA	−34.99872	151.02565	10	7	2	1	0
Tathra	TAT	−36.7381	149.9972	5	4	0	1	0
Indonesia/NT^a^	IND	−9.19671	129.29598	3	2	1	0	0
New Caledonia	NCA	−21.61646	165.36407	23	9	1	12	1
Norfolk Island	NFI	−29.04203	167.89909	18	1	1	12	4

^a^ Indonesia/NT = 5 × unknown sex, Whitsundays = 1 × unknown sex.

### 2.2 DNA Extraction & SNP Genotyping

Total genomic DNA was extracted from 10 to 15 mg tissue from each sample by Diversity Arrays Technologies (DArT Pty Ltd., Canberra, Australia) using a NucleoMag 96 Tissue Kit (Macherey‐Nagel, Düren, Germany) coupled with NucleoMag SEP to allow automated separation of high‐quality DNA on a Freedom Evo robotic liquid handler (TECAN, Männedorf, Switzerland). Single Nucleotide Polymorphism (SNP) genotyping was subsequently performed by combining DArT's proprietary genome complexity reduction method with next‐generation sequencing (Wenzl et al. 2004; Sansaloni et al. 2011; Kilian et al. 2012). Reduced genome complexity libraries for *G. cuvier* were generated including DNA digestion and ligation steps including a PstI/TaqI restriction enzyme combination with barcode adaptor sequences that allowed for selecting amplification of restriction fragments through 30 cycles of polymerase chain reaction (PCR) using DArT *PstI* primers (5′‐GAT GGA TCC AGT GCA G‐3′). The PCR conditions included an initial denaturation at 94°C for 1 min, followed by 30 cycles at 94°C for 20 s (denaturation), 58°C for 30 s (annealing), and 72°C for 45 s (extension), with a final extension at 72°C for 7 min. The amplified PCR products were sequenced on the HiSeq2500 platform (Illumina, San Diego, USA) using single‐read sequencing for 77 cycles, which yielding an average of 3448 million reads per sample. DArT's analytical pipeline (Ren et al. 2015; Egea et al. 2017) was subsequently used to demultiplex DNA sequences from individual samples, filter out poor‐quality sequences with Phred scores < 30, and to call SNP genotypes. Genotyping yielded a total of 32,690 SNP loci from 407 individual tiger sharks with an average of 20× sequence coverage. SNP loci were further filtered using the package dartR version 1.9.9.1 (Gruber et al. 2018), retaining only a single SNP per tag, removing secondaries, applying individual and locus call rate thresholds to 80%, reproducibility of 90%, and a minimum minor allele frequency of 5%. Finally, SNP loci deviating from Hardy–Weinberg expectations (*p* < 0.05) were removed from the dataset, and a hamming distance threshold of 0.2 was applied to control for potential paralogs. After filtering total of 10,709 SNP loci from 407 individual tiger sharks and 21 sampling locations remained for downstream population genomic analysis.

### 2.3 Tests for Overall Population Genetic Structure

SNP frequencies over all loci were contrasted between individuals from each location to determine patterns of overall genetic structure and population connectivity. The software *hierfstat* implemented in R (Goudet 2005) was used to calculate global and pairwise measures of population differentiation (*F*_ST_; Weir and Cockerham 1984) on filtered SNP loci. An analysis of molecular variation (AMOVA) was performed in the R package *poppr* (Kamvar et al. 2014) using pairwise *F*_ST_ as the distance measure, and partitioning variation among sample sites and within sample sites, with significance determined based on 999 permutations. Discriminant Analysis of Principal Components (DAPC) was performed using the *adegenet* software (Jombart 2008; Jombart and Ahmed 2011) implemented in the R package using the find clusters function. In addition, the Bayesian analysis package STRUCTURE (Pritchard et al. 2000) was used to test for overall population genetic structure. STRUCTURE was used to identify the number of distinct population clusters, to assign individuals to clusters, and to identify migrants and admixed individuals using genetic data only. To determine the number of population clusters (*K*), five independent simulations for *K* = 1–21 with 10,000 burn‐in and 100,000 data iterations were run. Analyses were performed using the admixture model of population structure (i.e., each individual draws some fraction of their genome from each of *K* populations) and allele frequencies were set as independent among populations. The most likely *K* was estimated using Evanno's Δ*K* (Evanno et al. 2005) in Structure Harvester (Earl and von Holdt 2012). Finally, relatedness estimates among all individuals were calculated using the R package SNPRelate (Zheng et al. 2012), with relatedness categories (first‐, second‐ and third‐degree relationships) inferred from co‐ancestry coefficients (*θ*) and accompanying *R*_0_ and *R*_1_ coefficients (Table S2).

### 2.4 Tests for Local Genetic Structure and Spatial Autocorrelation

Spatial autocorrelation analyses were performed in GenAlEx 6.51 (Peakall and Smouse 2006), providing a test of local genetic structuring and gene flow limitations among sampling locations. Analyses were performed on a random subset of 1000 SNP loci (due to computational limits of the program) with analyses performed on all sharks combined and subsequently sexes (male and female) and life stages separately (juvenile‐subadult and adult). Female sharks with a total length (TL) > 330 cm and males > 300 cm were considered adults, while those smaller were treated as juvenile‐subadults (as per *L*_50_ for this population, reported in Holmes et al. 2015). Distance classes for these analyses were based on the “equal sample size” option, with 10,000 permutations to test for levels of significance and using the “multi‐pop” test option. For each class, random permutations in the spatial locations of individuals (10,000 permutations) were then used to assess deviations of the relatedness coefficient (*R*) from 0. Distance classes were chosen so that they contained more than 100 pairwise comparisons, had a participation index > 50% and a coefficient of variation of participation of < 1 (Hardy and Vekemans 2002). The relatedness coefficient (*R*) was calculated for all pairs of individuals, with pairwise comparisons across eight distance classes ranging from 0 to 700 km. Deviation from 0 indicates that individuals within a given distance class are significantly more (positive values) or less (negative values) genetically similar than expected at random (Whiterod et al. 2016).

## 3 Results

### 3.1 Overall Population Genetic Structure

Population genomic analyses indicated weak but significant genetic structuring among sample locations (global *F*_ST_ = 0.001, 95% CIs: 0.001–0.002). This pattern was largely consistent across sharks varying by sex and life stage, with independent analyses indicating weak but significant genetic structuring in juvenile‐subadult females (global *F*_ST_ = 0.004, 95% CIs: 0.003–0.004), juvenile‐subadult males (global *F*_ST_ = 0.009, 95% CIs: 0.007–0.009), and adult females (global *F*_ST_ = 0.005, 95% CIs: 0.004–0.006). In contrast, a lack of significant genetic structure was observed in adult males (global *F*_ST_ = 0.000, 95% CIs: 0.000–0.001), although this estimate should be interpreted with caution due to sampling limitations. Overall, pairwise estimates of *F*_ST_ indicated weak genetic structure between sampling locations, with only 55 of 210 pairwise estimates differing significantly from zero (Table S2). AMOVA found no evidence for significant genetic differentiation between sample locations (0.12%, *p* > 0.001), with the majority of variance being explained by genetic variation between individuals within locations (99.88%, *p* < 0.001). Similarly, DAPC and STRUCTURE analyses indicated a lack of genetic structure, both identifying a single population cluster (*K* = 1, Δ*K* = 1; Figures S1 and S2). Finally, kinship estimates among all individuals generated in the SNPRelate package using multiple relatedness measures indicated low overall relatedness among shark pairs. From a total of 82,621 pairwise relatedness measures, > 99% were identified as unrelated individuals. Overall, only six first‐degree (full‐sibling), five second‐degree (half‐sibling), and 16 third‐degree (quarter‐sibling) relationships among shark pairs were recorded (Table S3). Related individuals were not associated with common sampling locations.

### 3.2 Local Genetic Structure and Spatial Autocorrelation

**FIGURE 2:**
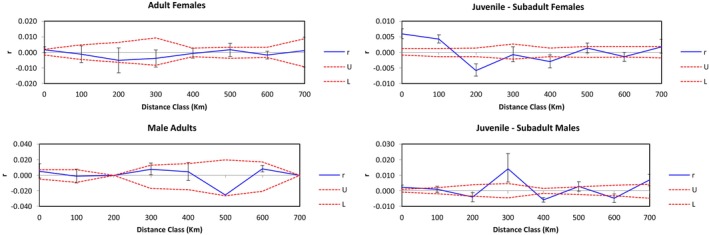
Spatial autocorrelation coefficient (*r*) for SNP data over a range of geographic distance classes spanning 700 km for adult females (top left), adult males (bottom left), juvenile‐subadult females (top right), and juvenile‐subadult males (bottom right).

Despite limited evidence of overall genetic structure, spatial autocorrelation analyses indicated local genetic structuring and gene flow limitations. The relatedness coefficient was calculated for all pairs of individuals, involving 80,200 pairwise comparisons across eight distance classes, ranging from 0 to 700 km. Significant and positive spatial autocorrelation was observed up to 100 km (Figure S3), suggesting that individuals at this spatial scale are more genetically similar than would be expected at random. Separate analyses performed for different sexes and life stages indicated that the signal of local genetic structure was driven by female juvenile‐subadult tiger sharks only. Here, significant and positive spatial autocorrelation was detected to a distance of 100 k based on 11,781 pairwise comparisons (Figure 2). This is further supported by a significant and negative pattern of spatial autocorrelation at 200 km, suggesting sharks at this spatial scale are less genetically similar than expected under a random mating scenario. Beyond 200 km, female juvenile‐subadult tiger sharks appear to be no more or less genetically similar than expected by chance. In contrast, spatial autocorrelation analyses of adult females (6786 pairwise comparisons) displayed no significant evidence of local genetic structuring (Figure 2). A lack of spatial autocorrelation was also observed when juvenile‐subadult males (4371 pairwise comparisons) were analyzed separately, with the exception of a single significant and positive signal observed at 300 km. However, this is unlikely to be biologically meaningful due to a lack of genetic structure at finer distance classes, and a relatively high degree of error around the mean based on a small number of pairwise comparisons for this distance class (94 pairwise comparisons). Finally, a lack of spatial autocorrelation was also observed in adult males (630 pairwise comparisons), although these outputs should be interpreted cautiously due to relatively small sample sizes (Figure 2, Table 2).

To test the effect of sampling size on the power for resolving signatures of local genetic structure, analyses were repeated using the female juvenile‐subadult tiger shark SNP dataset, subsampling 50 and 100 sharks three times at random and repeating the spatial autocorrelation analyses. In all cases, sample sizes of 50 failed to detect a signature of local genetic structure, while sample sizes of 100 successfully detected a consistent pattern of significant and positive spatial autocorrelation to a distance of 100 km (Figure S4). These findings provide confidence in our results suggesting a lack of local genetic structure in female adults (117 individuals) and male juvenile‐subadult (94 individuals) tiger sharks but suggest that tests for local genetic structure in male adults based on 36 individuals should be treated with caution.

## 4 Discussion

We demonstrate the importance of spatially and biologically (i.e., across sexes and life stages) replicated sampling regimes when characterizing the strength of gene flow and population connectivity in a large pelagic shark species. Our study points to weak genetic structuring in tiger sharks from eastern Australia and the Indo‐Pacific region, which supports findings from previous genetic studies that indicate the likely presence of a single panmictic population in the region (Bernard et al. 2016; Holmes et al. 2017). However, our stratified sampling regime along the east coast of Australia allowed for the detection of significant spatial autocorrelation and local genetic structuring in juvenile‐subadult female tiger sharks, indicating potential dispersal biases between sexes and life stages, and the possibility of female reproductive philopatry. These findings provide novel insights into the influence of both sex and ontogeny on patterns of population genetic structure and connectivity in eastern Australian tiger sharks, as well as essential habitats supporting the tiger shark population. Overall, this study highlights the importance of stratified sampling regimes and the risks of overstating the strength of biological connections among shark populations in the absence of appropriate sampling approaches.

### 4.1 Evidence of Fine‐Scale Genetic Structure

Our findings are consistent with previous studies that point to a lack of overall genetic structure among tiger sharks from eastern Australia and the Indo‐Pacific region (Bernard et al. 2016; Holmes et al. 2017). Tracking studies have also demonstrated potential for long‐distance migrations (over thousands of kilometers) in tiger sharks from this region, further supporting the notion of population panmixia (Lipscombe et al. 2020; Barnett et al. 2022). These findings are consistent with previous genetic and telemetry studies performed in the Atlantic Ocean, indicating population panmixia to be likely (Lea et al. 2015; Pirog, Jaquemet, et al. 2019; Sort et al. 2021; Hammerschlag et al. 2022). However, the strength of gene flow and connectivity within ocean basins remains uncertain due to sampling biases and limitations in most genetic and telemetry studies (Ferreira et al. 2015; Carmo et al. 2019; Pirog, Jaquemet, et al. 2019; Bernard et al. 2021; Hammerschlag et al. 2022). Studies have shown that patterns of genetic homogeneity and panmixia can be driven by low levels of gene flow and intergenerational migration between populations (Wright 1931; Whiterod et al. 2016). This potentially applies to tiger sharks given high levels of intraindividual variation in dispersal behaviors, including both large‐scale and restricted movement patterns, and evidence of potential sex‐ and ontogenetic‐biased dispersal (Driggers et al. 2008; Meyer et al. 2009; Werry et al. 2014; Ajemian et al. 2020; Barnett et al. 2022; McClain et al. 2022; Niella et al. 2022). Here, we demonstrate fine‐scale genetic structuring in juvenile‐subadult female tiger sharks from eastern Australia. These findings are most likely driven by demographic processes rather than spatially varying selection pressure, as candidate loci influenced by directional selection often persist in low abundance and have little effect on tests for overall population genetic structure (Holland et al. 2022; Sandoval‐Castillo et al. 2018). These findings support those of McClain et al. (2022) who recently reported size‐dependent genetic structuring in tiger sharks from the north‐western Atlantic Ocean, where genetic structure was greatest in juvenile and subadult life stages regardless of sex. Our findings build on those from McClain et al. (2022) suggesting that both sex and ontogeny are likely to be influencing patterns of genetic structure and population connectivity across the species range.

Our sampling approach enabled us to gain insight into the influence of sex and ontogeny on biological connections in tiger sharks from eastern Australia and the Indo‐Pacific region. Previous tracking studies from the same region have demonstrated year‐round residency in some juvenile‐subadult females in parts of eastern Australia and the Indo‐Pacific (Werry et al. 2014). However, most studies have reported movements of juvenile‐subadult females to be highly variable, with some individuals traveling over a thousand kilometers in ~30 days from the tagging event (Holmes et al. 2014; Meyer et al. 2009; Niella et al. 2022; Papastamatiou et al. 2013). Furthermore, a recent telemetry study indicated the degree of tiger shark residency to vary among individuals and regions in eastern Australia (Niella et al. 2022). Specifically, Niella et al. (2022) reported behavioral differences between tiger sharks from different tagging locations, where sharks from the northern extent of the coastline showed a greater degree of local residency than those from the central coast where movement patterns were more extensive. Our findings point to potential residency in juvenile‐subadult female tiger sharks, a spatial pattern which appears to be consistent across our sampling distribution in eastern Australia. However, evidence for substantial interindividual variability relating to both vagility and residency suggests that signatures of spatial autocorrelation might be driven by just a fraction of the juvenile‐subadult female tiger shark subpopulation. Further research is prudent to help validate these findings and to quantify the degree of residency in juvenile‐subadult female tiger sharks more accurately through more comprehensive tracking studies. Such efforts are expected to provide novel insights into essential habitats supporting tiger sharks in eastern Australia and potentially more widely across the Indo‐Pacific region.

In contrast, our results indicate a lack of genetic structure at both local and broad spatial scales in both male and female adult and male juvenile‐subadult tiger sharks. Although our results relating to male adult tiger sharks should be interpreted with some degree of caution due to sampling limitations, telemetry studies in eastern Australia and the Indo‐Pacific regions (and from other ocean basins) point to both male and female adult tiger sharks as being highly dispersive (Holmes et al. 2014; Ferreira et al. 2015; Lipscombe et al. 2020). In contrast, telemetry studies on male juvenile‐subadult tiger sharks have been less replicated, although they also suggest similar broad offshore movements and an ability to undertake deep dives to ~1 km (Lipscombe et al. 2020). In Hawaii, Meyer et al. (2009) also reported juveniles as significantly wider ranging than adults, perhaps driven by predation avoidance behaviors. As such, it remains difficult to determine any consistency in the movement drivers of tiger sharks, and we therefore advise that future studies should incorporate multiple lines of investigation including sex, ontogeny, and even differences in habitat use by this species (Vaudo et al. 2014; Lubitz et al. 2022).

### 4.2 Essential Habitats and Future Tiger Shark Research

Essential habitats are areas that support specific functions over various life‐history stages of a species life cycle, such as foraging, refuge, or reproductive purposes (Barnett et al. 2019; Heupel et al. 2019; De Wysiecki et al. 2023). Information on essential habitats that support the eastern Australian tiger shark population remains limited, including locations of natal habitats. Here, evidence of local genetic structuring, limited movement, and potential residency in juvenile‐subadult female tiger sharks is one of the earliest indications of potential reproductive philopatry and preferred parturition areas in tiger sharks from eastern Australia. At present, information on reproductive philopatry, and the behavior and habitat use of neonate and juvenile‐subadult tiger sharks (including their dispersal from birthing sites), is limited for this region (Holland et al. 2019). Evidence of tiger shark residency has been reported in some parts of the northern hemisphere, and linked to natal habitats, refuge behaviors, foraging, and reproductive philopatry (Driggers et al. 2008; Sulikowski et al. 2016; Acuña‐Marrero et al. 2017; McClain et al. 2022; Smukall et al. 2022). Furthermore, it has been suggested that some oceanic islands are essential to the reproductive cycle of tiger sharks, including the Bahamas (Sulikowski et al. 2016; Smukall et al. 2022), Hawaii (Whitney and Crow 2007; Papastamatiou et al. 2013), Galapagos Islands (Acuña‐Marrero et al. 2017), and Cocos Island (Cambra et al. 2021), and possibly Norfolk Island (Matley et al. 2024). Our findings point to the possibility of female reproductive philopatry in coastal or shelf regions of eastern Australia, with natal habitats potentially spanning most of the coastline from tropical to temperate waters. However, it is unknown if parturition is likely to be occurring at specific natal grounds/nursery areas, or in “preferred” general areas spanning 10s of kms, which seems most probable given the scale of spatial autocorrelation observed in the current study.

Additional research is required to validate these findings. For example, investigating animal movement and habitat use employing a similar stratified sampling design as the current study. Although Niella et al. (2022) did not detect significant effects of sex or ontogeny on tiger shark dispersal patterns in this region, more replicated sampling is needed to confirm these findings. Furthermore, an expansion of sampling to include neonate and small juveniles will help to detect the degree of genetic structure in earlier life stages and determine the likely locations of natal habitats in the region. However, accessing and sampling these size ranges remains challenging as encounters are rare using commonly used survey gear (Holmes et al. 2014; Niella et al. 2022; Tate et al. 2021). Alternatively, it has been previously suggested that fitting females confirmed through ultrasound to be pregnant (Sulikowski et al. 2024) with intrauterine transmitters (Sulikowski and Hammerschlag 2023) can help to detect pupping events and locations, which could assist with future genetic sampling of early life stages.

### 4.3 Importance of Stratified Sampling

This study adds to a growing body of literature supporting the importance of stratified sampling regimes when resolving patterns of population genetic structure and connectivity in elasmobranchs. Phillips et al. (2021) recently challenged the integrity of elasmobranch population genetic studies, arguing that the dependency on opportunistic sampling regimes that leads to the pooling of animals varying in sex and life stages is likely to be obscuring genetic signals of sex‐biased dispersal and reproductive philopatry. Our study supports this argument, demonstrating that the pooling of samples can obscure patterns of genetic structure associated with specific sexes and life stages. Furthermore, we demonstrate the importance of spatially replicated sampling regimes that allow for direct tests of spatial autocorrelation and provide insights into the true strength and spatial limits of gene flow. Consequently, it is possible that the strength of biological connectivity among elasmobranchs both within and across the world's oceans has been overstated due to the general reliance of previous genetic studies on nonstratified sampling approaches (Table 1).

Improving sampling design is required for future genetic studies on elasmobranchs. However, as discussed by Phillips et al. (2021), this often requires highly collaborative research and significant resourcing. Sampling efforts for the current study were made possible by several research programs geared toward mitigating risks of human–shark interactions in eastern Australia and the Indo‐Pacific region. However, this type of sampling requires a high level of investment and may not be possible in other parts of the world due to logistical and financial constraints, or in study systems with low population abundances (e.g., rare and threatened species). Consequently, researchers and managers should be cautious when interpreting patterns of population genetic structure and connectivity in elasmobranchs in the absence of stratified sampling regimes, as this could risk misleading conservation and management decision‐making processes.

## Conflicts of Interest

The authors declare no conflicts of interest.

## Supporting information


Data S1

## Data Availability

Files containing filtered SNPs used for population genomic analyses are deposited in the Zenodo archive for samples from New Caledonia (https://doi.org/10.5281/zenodo.14279842) and in the Dryad archive for all other samples (https://doi.org/10.5061/dryad.1rn8pk128).
