# Supplementary material for: Evidence of Fine‐Scale Genetic Structure in Tiger Sharks (Galeocerdo cuvier) Highlights the Importance of Stratified Sampling Regimes
Source: Evol Appl. 2025 Jun 8;18(6):e70117. doi: 10.1111/eva.70117 (PMC12146213; doi:10.1111/eva.70117)
Supplement: Supplementary file 1 — Data S1 [file EVA-18-e70117-s001.docx]

**Supplementary Material**

**Evidence of fine-scale genetic structure in tiger sharks (*Galeocerdo cuvier*) highlights the importance of stratified sampling regimes**

Jessica J. Fish^1^, Christine Dudgeon^2^, Adam Barnett^3,4^, Paul A. Butcher^1,5^, Bonnie J. Holmes^6^, Charlie Huveneers^7^, Lauren Meyer^7^, Laurent Vigliola^8^, Craig D.H Sherman^1^, Adam D. Miller^1^

^1^ Deakin University School of Life and Environmental Sciences, Warrnambool, VIC, Australia

^2^ University of the Sunshine Coast, School of Science, Technology & Engineering, Moreton, QLD, Australia

^3^ Biopixel Oceans Foundation, Cairns, Qld

^4^ Marine Data Technology Hub, James Cook University, Townsville, QLD, Australia

^5^ New South Wales Department of Primary Industries, National Marine Science Centre, Coffs Harbour, NSW, Australia

^6^ University of the Sunshine Coast, School of Science, Technology & Engineering, Sippy Downs, QLD, Australia

^7^ Flinders University, College of Science and Engineering, Adelaide, SA, Australia

^8^ ENTROPIE, Institut de Recherche pour le Développement, Noumea, New Caledonia, France

**Table S1.** Details on individual sharks from which tissue biopsies were used for population genomic analyses.

| Date sampled | Shark number | Sex | TL (cm) | Location caught | Latitude | Longitude |
| --- | --- | --- | --- | --- | --- | --- |
| 18/10/2019 | 100T | F | 201 | Ballina | -28.8329 | 153.6102 |
| 28/10/2019 | 101T | F | 301 | Ballina | -28.8329 | 153.6102 |
| 8/11/2019 | 104T | M | 302 | Ballina | -28.8329 | 153.6102 |
| 11/12/2019 | 106T | M | 199 | Ballina | -28.8329 | 153.6102 |
| 13/12/2019 | 108T | M | 245 | Ballina | -28.8329 | 153.6102 |
| 27/12/2019 | 109T | M | 255 | Ballina | -28.8329 | 153.6102 |
| 12/01/2020 | 110T | F | 229 | Ballina | -28.8329 | 153.6102 |
| 24/01/2020 | 111T | M | 233 | Ballina | -28.8329 | 153.6102 |
| 22/02/2020 | 112T | F | 361 | Ballina | -28.8329 | 153.6102 |
| 26/02/2020 | 113T | F | 330 | Ballina | -28.8329 | 153.6102 |
| 5/03/2020 | 114T | F | 251 | Ballina | -28.8329 | 153.6102 |
| 5/03/2020 | 115T | M | 281 | Ballina | -28.8329 | 153.6102 |
| 5/03/2020 | 116T | M | 154 | Ballina | -28.8329 | 153.6102 |
| 5/03/2020 | 117T | F | 272 | Ballina | -28.8329 | 153.6102 |
| 6/03/2020 | 118T | M | 192 | Ballina | -28.8329 | 153.6102 |
| 24/03/2020 | 120T | M | 283 | Ballina | -28.8329 | 153.6102 |
| 25/03/2020 | 121T | M | 208 | Ballina | -28.8329 | 153.6102 |
| 26/03/2020 | 123T | F | 246 | Ballina | -28.8329 | 153.6102 |
| 26/03/2020 | 124T | M | 252 | Ballina | -28.8329 | 153.6102 |
| 31/03/2020 | 125T | F | 201 | Ballina | -28.8329 | 153.6102 |
| 2/04/2020 | 127T | F | 238 | Ballina | -28.8329 | 153.6102 |
| 2/04/2020 | 129T | M | 283 | Ballina | -28.8329 | 153.6102 |
| 2/04/2020 | 130T | M | 214 | Ballina | -28.8329 | 153.6102 |
| 3/04/2020 | 131T | M | 207 | Ballina | -28.8329 | 153.6102 |
| 17/04/2020 | 133T | M | 260 | Ballina | -28.8329 | 153.6102 |
| 3/05/2020 | 136T | F | 226 | Ballina | -28.8329 | 153.6102 |
| 30/06/2020 | 137T | F | 289 | Ballina | -28.8329 | 153.6102 |
| 23/07/2020 | 138T | F | 268 | Ballina | -28.8329 | 153.6102 |
| 24/07/2020 | 139T | M | 248 | Ballina | -28.8329 | 153.6102 |
| 4/09/2020 | 140T | M | 167 | Ballina | -28.8329 | 153.6102 |
| 10/10/2020 | 141T | F | 270 | Ballina | -28.8329 | 153.6102 |
| 19/10/2020 | 143T | F | 227 | Ballina | -28.8329 | 153.6102 |
| 17/11/2020 | 153T | F | 226 | Ballina | -28.8329 | 153.6102 |
| 17/11/2020 | 154T | F | 217 | Ballina | -28.8329 | 153.6102 |
| 19/11/2020 | 155T | F | 261 | Ballina | -28.8329 | 153.6102 |
| 19/11/2020 | 156T | M | 253 | Ballina | -28.8329 | 153.6102 |
| 22/11/2020 | 157T | F | 271 | Ballina | -28.8329 | 153.6102 |
| 22/11/2020 | 158T | F | 289 | Ballina | -28.8329 | 153.6102 |
| 7/12/2020 | 159T | F | 199 | Ballina | -28.8329 | 153.6102 |
| 8/12/2020 | 160T | F | 139 | Ballina | -28.8329 | 153.6102 |
| 3/01/2021 | 162T | F | 291 | Ballina | -28.8329 | 153.6102 |
| 17/01/2021 | 163T | M | 257 | Ballina | -28.8329 | 153.6102 |
| 13/02/2021 | 164T | F | 202 | Ballina | -28.8329 | 153.6102 |
| 23/02/2021 | 165T | F | 203 | Ballina | -28.8329 | 153.6102 |
| 5/03/2021 | 167T | M | 260 | Ballina | -28.8329 | 153.6102 |
| 29/04/2021 | 170T | M | 219 | Ballina | -28.8329 | 153.6102 |
| 29/04/2021 | 171T | M | 256 | Ballina | -28.8329 | 153.6102 |
| 29/04/2021 | 172T | M | 273 | Ballina | -28.8329 | 153.6102 |
| 24/05/2021 | 174T | F | 348 | Ballina | -28.8329 | 153.6102 |
| 7/06/2021 | 176T | F | 251 | Ballina | -28.8329 | 153.6102 |
| 12/06/2021 | 178T | M | 250 | Ballina | -28.8329 | 153.6102 |
| 6/01/2018 | 21T | M | 212 | Ballina | -28.8329 | 153.6102 |
| 15/02/2018 | 35T | M | 215 | Ballina | -28.8329 | 153.6102 |
| 4/03/2018 | 40T | M | 160 | Ballina | -28.8329 | 153.6102 |
| 25/07/2018 | 46T | F | 394 | Ballina | -28.8329 | 153.6102 |
| 22/08/2018 | 47T | M | 178 | Ballina | -28.8329 | 153.6102 |
| 9/09/2018 | 48T | F | 192 | Ballina | -28.8329 | 153.6102 |
| 31/10/2018 | 49T | F | 270 | Ballina | -28.8329 | 153.6102 |
| 22/12/2018 | 51T | M | 282 | Ballina | -28.8329 | 153.6102 |
| 1/01/2019 | 52T | F | 287 | Ballina | -28.8329 | 153.6102 |
| 23/01/2019 | 53T | F | 226 | Ballina | -28.8329 | 153.6102 |
| 4/02/2019 | 56T | F | 280 | Ballina | -28.8329 | 153.6102 |
| 7/02/2019 | 57T | F | 261 | Ballina | -28.8329 | 153.6102 |
| 20/02/2019 | 58T | F | 235 | Ballina | -28.8329 | 153.6102 |
| 20/02/2019 | 59T | M | 266 | Ballina | -28.8329 | 153.6102 |
| 16/03/2019 | 63T | M | 254 | Ballina | -28.8329 | 153.6102 |
| 20/03/2019 | 64T | M | 239 | Ballina | -28.8329 | 153.6102 |
| 22/03/2019 | 65T | M | 264 | Ballina | -28.8329 | 153.6102 |
| 24/03/2019 | 66T | M | 230 | Ballina | -28.8329 | 153.6102 |
| 24/04/2019 | 79T | F | 223 | Ballina | -28.8329 | 153.6102 |
| 11/06/2019 | 92T | F | 354 | Ballina | -28.8329 | 153.6102 |
| 9/07/2019 | 93T | F | 384 | Ballina | -28.8329 | 153.6102 |
| 20/07/2019 | 95T | F | 179 | Ballina | -28.8329 | 153.6102 |
| 23/07/2019 | 96T | F | 330 | Ballina | -28.8329 | 153.6102 |
| 26/09/2019 | 97T | F | 308 | Ballina | -28.8329 | 153.6102 |
| 12/10/2019 | 98T | F | 218 | Ballina | -28.8329 | 153.6102 |
| 14/10/2019 | 99T | M | 196 | Ballina | -28.8329 | 153.6102 |
| NA | 2CD | F | 355 | Bundaberg | -24.60315 | 152.85571 |
| NA | 3CD | F | 344 | Bundaberg | -24.60315 | 152.85571 |
| NA | 4CD | F | 301 | Bundaberg | -24.60315 | 152.85571 |
| NA | TS002 | M | 110 | Bundaberg | -24.60315 | 152.85571 |
| NA | TS004 | F | 101 | Bundaberg | -24.60315 | 152.85571 |
| NA | TS006 | M | 330 | Bundaberg | -24.60315 | 152.85571 |
| NA | TS008 | M | 253 | Bundaberg | -24.60315 | 152.85571 |
| NA | TS011 | F | 310 | Bundaberg | -24.60315 | 152.85571 |
| NA | TS013 | F | 247 | Bundaberg | -24.60315 | 152.85571 |
| NA | TS014 | F | 370 | Bundaberg | -24.60315 | 152.85571 |
| NA | TS015 | M | 220 | Bundaberg | -24.60315 | 152.85571 |
| NA | TS016 | F | 257 | Bundaberg | -24.60315 | 152.85571 |
| NA | TS021 | M | 118 | Bundaberg | -24.60315 | 152.85571 |
| NA | TS024 | M | 167 | Bundaberg | -24.60315 | 152.85571 |
| NA | TS026 | M | 130 | Bundaberg | -24.60315 | 152.85571 |
| NA | TS027 | M | 135 | Bundaberg | -24.60315 | 152.85571 |
| NA | TS028 | F | 202 | Bundaberg | -24.60315 | 152.85571 |
| NA | TS029 | F | 360 | Bundaberg | -24.60315 | 152.85571 |
| NA | TS031 | M | 178 | Bundaberg | -24.60315 | 152.85571 |
| NA | TS032 | M | 129 | Bundaberg | -24.60315 | 152.85571 |
| NA | TS034 | F | 109 | Bundaberg | -24.60315 | 152.85571 |
| NA | TS035 | F | 154 | Bundaberg | -24.60315 | 152.85571 |
| NA | TS038 | M | 146 | Bundaberg | -24.60315 | 152.85571 |
| NA | TS039 | F | 195 | Bundaberg | -24.60315 | 152.85571 |
| NA | TS042 | M | 188 | Bundaberg | -24.60315 | 152.85571 |
| NA | TS043 | F | 210 | Bundaberg | -24.60315 | 152.85571 |
| NA | TS044 | M | 270 | Bundaberg | -24.60315 | 152.85571 |
| NA | TS047 | F | 310 | Bundaberg | -24.60315 | 152.85571 |
| NA | TS047CD | F | 310 | Bundaberg | -24.60315 | 152.85571 |
| NA | 10CD | M | 228 | Cairns | -16.74665 | 145.87043 |
| NA | 5CD | F | 210 | Cairns | -16.74665 | 145.87043 |
| NA | 6CD | F | 360 | Cairns | -16.74665 | 145.87043 |
| NA | 7CD | F | 317 | Cairns | -16.74665 | 145.87043 |
| NA | 8CD | F | 292 | Cairns | -16.74665 | 145.87043 |
| NA | 9CD | F | 322 | Cairns | -16.74665 | 145.87043 |
| 23/10/2018 | B10 | F | 285 | Cairns | -16.74665 | 145.87043 |
| 23/10/2018 | B11 | F | 231 | Cairns | -16.74665 | 145.87043 |
| 23/10/2018 | B12 | F | 262 | Cairns | -16.74665 | 145.87043 |
| 12/08/2015 | B2 | F | 260 | Cairns | -16.74665 | 145.87043 |
| 24/07/2017 | B5 | F | 240 | Cairns | -16.74665 | 145.87043 |
| 25/07/2017 | B6 | F | 350 | Cairns | -16.74665 | 145.87043 |
| 25/07/2017 | B7 | F | 250 | Cairns | -16.74665 | 145.87043 |
| 22/10/2018 | B8 | F | 240 | Cairns | -16.74665 | 145.87043 |
| 23/10/2018 | B9 | F | 305 | Cairns | -16.74665 | 145.87043 |
| 1/12/2020 | CN1 | F | 300 | Cairns | -16.74665 | 145.87043 |
| 2/12/2020 | CN2 | M | 205 | Cairns | -16.74665 | 145.87043 |
| NA | TS045 | M | 156 | Cairns | -16.74665 | 145.87043 |
| 16/03/2021 | GBR1 | F | 376 | Capricorn Coast | -23.295484 | 151.69477 |
| 16/03/2021 | GBR2 | F | 267 | Capricorn Coast | -23.295484 | 151.69477 |
| 16/03/2021 | GBR3 | F | 302 | Capricorn Coast | -23.295484 | 151.69477 |
| 16/03/2021 | GBR4 | F | 272 | Capricorn Coast | -23.295484 | 151.69477 |
| 16/03/2021 | GBR5 | F | 383 | Capricorn Coast | -23.295484 | 151.69477 |
| NA | TS085 | F | 92 | Capricorn Coast | -23.295484 | 151.69477 |
| 30/10/2017 | 11T | M | 191 | Coffs Harbour | -30.2922 | 153.154 |
| 14/11/2017 | 13T | F | 184 | Coffs Harbour | -30.2922 | 153.154 |
| 15/11/2017 | 14T | F | 203 | Coffs Harbour | -30.2922 | 153.154 |
| 16/11/2017 | 15T | F | 174 | Coffs Harbour | -30.2922 | 153.154 |
| 29/11/2017 | 16T | F | 210 | Coffs Harbour | -30.2922 | 153.154 |
| 6/01/2018 | 20T | F | 238 | Coffs Harbour | -30.2922 | 153.154 |
| 10/01/2018 | 22T | F | 212 | Coffs Harbour | -30.2922 | 153.154 |
| 11/01/2018 | 23T | F | 205 | Coffs Harbour | -30.2922 | 153.154 |
| 12/01/2018 | 24T | M | 182 | Coffs Harbour | -30.2922 | 153.154 |
| 27/01/2018 | 25T | F | 206 | Coffs Harbour | -30.2922 | 153.154 |
| 28/01/2018 | 26T | F | 212 | Coffs Harbour | -30.2922 | 153.154 |
| 29/01/2018 | 27T | F | 221 | Coffs Harbour | -30.2922 | 153.154 |
| 29/01/2018 | 28T | F | 210 | Coffs Harbour | -30.2922 | 153.154 |
| 30/01/2018 | 29T | F | 221 | Coffs Harbour | -30.2922 | 153.154 |
| 13/02/2018 | 32T | F | 188 | Coffs Harbour | -30.2922 | 153.154 |
| 13/02/2018 | 33T | F | 242 | Coffs Harbour | -30.2922 | 153.154 |
| 25/01/2019 | 55T | M | 248 | Coffs Harbour | -30.2922 | 153.154 |
| 29/03/2019 | 68T | F | 251 | Coffs Harbour | -30.2922 | 153.154 |
| 29/03/2019 | 69T | F | 216 | Coffs Harbour | -30.2922 | 153.154 |
| 25/04/2019 | 80T | F | 160 | Coffs Harbour | -30.2922 | 153.154 |
| 8/05/2019 | 83T | M | 157 | Coffs Harbour | -30.2922 | 153.154 |
| 8/05/2019 | 84T | F | 236 | Coffs Harbour | -30.2922 | 153.154 |
| 9/05/2019 | 85T | M | 284 | Coffs Harbour | -30.2922 | 153.154 |
| 9/05/2019 | 86T | M | 176 | Coffs Harbour | -30.2922 | 153.154 |
| 14/05/2019 | 88T | F | 228 | Coffs Harbour | -30.2922 | 153.154 |
| 14/05/2019 | 89T | F | 212 | Coffs Harbour | -30.2922 | 153.154 |
| 6/09/2017 | 8T | F | 191 | Coffs Harbour | -30.2922 | 153.154 |
| 16/09/2017 | 9T | F | 159 | Coffs Harbour | -30.2922 | 153.154 |
| 11/12/2019 | 107T | F | 193 | Evans Head | -29.1104 | 153.4412 |
| 2/04/2020 | 128T | M | 253 | Evans Head | -29.1104 | 153.4412 |
| 7/04/2020 | 132T | F | 215 | Evans Head | -29.1104 | 153.4412 |
| 22/04/2020 | 134T | F | 287 | Evans Head | -29.1104 | 153.4412 |
| 1/05/2020 | 135T | F | 245 | Evans Head | -29.1104 | 153.4412 |
| 14/03/2021 | 168T | M | 270 | Evans Head | -29.1104 | 153.4412 |
| 9/06/2021 | 177T | F | 245 | Evans Head | -29.1104 | 153.4412 |
| 18/06/2018 | 45T | F | 204 | Evans Head | -29.1104 | 153.4412 |
| 23/01/2019 | 54T | M | 234 | Evans Head | -29.1104 | 153.4412 |
| 29/03/2017 | 5T | F | 241 | Evans Head | -29.1104 | 153.4412 |
| 15/03/2019 | 62T | M | 263 | Evans Head | -29.1104 | 153.4412 |
| 5/04/2019 | 71T | F | 354 | Evans Head | -29.1104 | 153.4412 |
| 13/04/2019 | 74T | M | 202 | Evans Head | -29.1104 | 153.4412 |
| 15/04/2019 | 75T | F | 201 | Evans Head | -29.1104 | 153.4412 |
| 15/04/2019 | 76T | F | 203 | Evans Head | -29.1104 | 153.4412 |
| 17/04/2019 | 77T | M | 232 | Evans Head | -29.1104 | 153.4412 |
| 17/04/2019 | 78T | M | 187 | Evans Head | -29.1104 | 153.4412 |
| 2/05/2019 | 81T | F | 197 | Evans Head | -29.1104 | 153.4412 |
| 7/05/2019 | 82T | F | 194 | Evans Head | -29.1104 | 153.4412 |
| 17/05/2019 | 90T | F | 227 | Evans Head | -29.1104 | 153.4412 |
| 25/05/2019 | 91T | M | 167 | Evans Head | -29.1104 | 153.4412 |
| 13/07/2019 | 94T | F | 364 | Evans Head | -29.1104 | 153.4412 |
| NA | TS245 | F | 139 | Evans Head | -29.1104 | 153.4412 |
| NA | TS252 | M | 128 | Evans Head | -29.1104 | 153.4412 |
| NA | TS253 | M | 169 | Evans Head | -29.1104 | 153.4412 |
| NA | TS048 | F | 260 | Gold Coast | -27.94874 | 153.55313 |
| NA | TS049 | M | 270 | Gold Coast | -27.94874 | 153.55313 |
| NA | TS050 | F | 380 | Gold Coast | -27.94874 | 153.55313 |
| NA | TS103 | M | 145 | Gold Coast | -27.94874 | 153.55313 |
| NA | TS104 | F | 240 | Gold Coast | -27.94874 | 153.55313 |
| NA | TS105 | M | 141 | Gold Coast | -27.94874 | 153.55313 |
| 1/04/2015 | 10AB | M | 230 | Indo NT | -9.19671 | 129.29598 |
| NA | TS412 | NA | NA | Indo NT | -9.19671 | 129.29598 |
| NA | TS433 | F | NA | Indo NT | -9.19671 | 129.29598 |
| NA | TS434 | F | NA | Indo NT | -9.19671 | 129.29598 |
| NA | TS466 | NA | NA | Indo NT | -9.19671 | 129.29598 |
| NA | TS469 | NA | NA | Indo NT | -9.19671 | 129.29598 |
| NA | TS470 | NA | NA | Indo NT | -9.19671 | 129.29598 |
| NA | TS471 | NA | NA | Indo NT | -9.19671 | 129.29598 |
| 9/12/2017 | 17T | F | 189 | Kiama | -34.99872 | 151.02565 |
| 13/12/2017 | 18T | F | 375 | Kiama | -34.99872 | 151.02565 |
| 15/12/2017 | 19T | F | 175 | Kiama | -34.99872 | 151.02565 |
| 7/02/2018 | 30T | M | 186 | Kiama | -34.99872 | 151.02565 |
| 21/02/2018 | 36T | F | 156 | Kiama | -34.99872 | 151.02565 |
| 3/03/2018 | 39T | F | 192 | Kiama | -34.99872 | 151.02565 |
| 8/03/2018 | 41T | F | 193 | Kiama | -34.99872 | 151.02565 |
| 15/04/2018 | 42T | M | 242 | Kiama | -34.99872 | 151.02565 |
| 16/04/2018 | 43T | F | 192 | Kiama | -34.99872 | 151.02565 |
| 17/04/2018 | 44T | F | 171 | Kiama | -34.99872 | 151.02565 |
| 13/02/2018 | 34T | F | 149 | Laurieton | -31.71084 | 152.98571 |
| NA | TS226 | M | 273 | Laurieton | -31.71084 | 152.98571 |
| NA | TS227 | F | 266 | Laurieton | -31.71084 | 152.98571 |
| NA | TS228 | F | 217 | Laurieton | -31.71084 | 152.98571 |
| NA | TS229 | F | 380 | Laurieton | -31.71084 | 152.98571 |
| NA | TS230 | F | 180 | Laurieton | -31.71084 | 152.98571 |
| NA | TS087 | F | 196 | Mackay | -21.13224 | 149.29475 |
| NA | TS088 | F | 111 | Mackay | -21.13224 | 149.29475 |
| NA | TS089 | M | 165 | Mackay | -21.13224 | 149.29475 |
| NA | TS090 | F | 258 | Mackay | -21.13224 | 149.29475 |
| NA | TS091 | F | NA | Mackay | -21.13224 | 149.29475 |
| NA | TS092 | F | 227 | Mackay | -21.13224 | 149.29475 |
| NA | TS093 | F | 276 | Mackay | -21.13224 | 149.29475 |
| NA | TS094 | F | 200 | Mackay | -21.13224 | 149.29475 |
| NA | TS095 | F | 187 | Mackay | -21.13224 | 149.29475 |
| NA | TS096 | F | 194 | Mackay | -21.13224 | 149.29475 |
| NA | TS097 | F | 180 | Mackay | -21.13224 | 149.29475 |
| NA | TS098 | M | 242 | Mackay | -21.13224 | 149.29475 |
| NA | TS099 | M | 208 | Mackay | -21.13224 | 149.29475 |
| NA | TS100 | F | 226 | Mackay | -21.13224 | 149.29475 |
| NA | TS101 | F | 390 | Mackay | -21.13224 | 149.29475 |
| NA | TS102 | M | 200 | Mackay | -21.13224 | 149.29475 |
| 4/03/2021 | NCB007 | F | 294 | New Caledonia | -21.616464 | 165.364073 |
| 12/03/2021 | NCB022 | F | 396 | New Caledonia | -21.616464 | 165.364073 |
| 19/11/2020 | NCB024 | F | 148 | New Caledonia | -21.616464 | 165.364073 |
| 12/03/2021 | NCB026 | F | 386 | New Caledonia | -21.616464 | 165.364073 |
| 12/03/2021 | NCB027 | F | 361 | New Caledonia | -21.616464 | 165.364073 |
| 25/03/2021 | NCB029 | F | 312 | New Caledonia | -21.616464 | 165.364073 |
| 25/03/2021 | NCB039 | F | 281 | New Caledonia | -21.616464 | 165.364073 |
| 25/03/2021 | NCB043 | F | 274 | New Caledonia | -21.616464 | 165.364073 |
| 17/03/2021 | NCB044 | F | 298 | New Caledonia | -21.616464 | 165.364073 |
| 4/03/2021 | NCB045 | F | 376 | New Caledonia | -21.616464 | 165.364073 |
| 12/03/2021 | NCB050 | M | 160 | New Caledonia | -21.616464 | 165.364073 |
| 28/04/2021 | NCB052 | F | 157 | New Caledonia | -21.616464 | 165.364073 |
| 28/04/2021 | NCB053 | F | 375 | New Caledonia | -21.616464 | 165.364073 |
| 19/11/2020 | NCB056 | F | 220 | New Caledonia | -21.616464 | 165.364073 |
| 27/07/2021 | NCB057 | F | 370 | New Caledonia | -21.616464 | 165.364073 |
| 27/07/2021 | NCB058 | F | 300 | New Caledonia | -21.616464 | 165.364073 |
| 27/07/2021 | NCB059 | F | 168 | New Caledonia | -21.616464 | 165.364073 |
| 28/04/2021 | NCB060 | F | 245 | New Caledonia | -21.616464 | 165.364073 |
| 15/04/2016 | NCB063 | F | 253 | New Caledonia | -21.616464 | 165.364073 |
| 18/04/2016 | NCB064 | M | 280 | New Caledonia | -21.616464 | 165.364073 |
| 4/05/2016 | NCB065 | F | 270 | New Caledonia | -21.616464 | 165.364073 |
| 5/05/2016 | NCB066 | F | 373 | New Caledonia | -21.616464 | 165.364073 |
| 5/06/2016 | NCB067 | F | 250 | New Caledonia | -21.616464 | 165.364073 |
| 25/02/2020 | NFI17 | M | NA | Norfolk Island | -29.04203 | 167.89909 |
| 26/02/2020 | NFI18 | F | 408 | Norfolk Island | -29.04203 | 167.89909 |
| 27/02/2020 | NFI20 | M | 382 | Norfolk Island | -29.04203 | 167.89909 |
| 27/02/2020 | NFI21 | M | 396 | Norfolk Island | -29.04203 | 167.89909 |
| 28/02/2020 | NFI24 | F | 363 | Norfolk Island | -29.04203 | 167.89909 |
| 28/02/2020 | NFI25 | F | 314 | Norfolk Island | -29.04203 | 167.89909 |
| 28/02/2020 | NFI26 | M | 405 | Norfolk Island | -29.04203 | 167.89909 |
| 24/02/2021 | NFI32 | F | 370 | Norfolk Island | -29.04203 | 167.89909 |
| 24/02/2021 | NFI33 | F | 358 | Norfolk Island | -29.04203 | 167.89909 |
| 24/02/2021 | NFI34 | F | 390 | Norfolk Island | -29.04203 | 167.89909 |
| 24/02/2021 | NFI35 | F | 400 | Norfolk Island | -29.04203 | 167.89909 |
| 24/02/2021 | NFI36 | F | 417 | Norfolk Island | -29.04203 | 167.89909 |
| 24/02/2021 | NFI37 | M | 372 | Norfolk Island | -29.04203 | 167.89909 |
| 26/02/2021 | NFI39 | F | 384 | Norfolk Island | -29.04203 | 167.89909 |
| 26/02/2021 | NFI40 | F | 424 | Norfolk Island | -29.04203 | 167.89909 |
| 26/02/2021 | NFI41 | F | 400 | Norfolk Island | -29.04203 | 167.89909 |
| 19/02/2020 | NFI5 | F | 380 | Norfolk Island | -29.04203 | 167.89909 |
| 26/02/2020 | NFI6 | F | NA | Norfolk Island | -29.04203 | 167.89909 |
| 24/08/2021 | Hinch13 | M | 300 | Palm Islands | -18.65251 | 146.59898 |
| 18/04/2021 | OI003 | F | 145 | Palm Islands | -18.65251 | 146.59898 |
| 24/06/2021 | OI013 | M | 316 | Palm Islands | -18.65251 | 146.59898 |
| 25/06/2021 | OI019 | F | 110 | Palm Islands | -18.65251 | 146.59898 |
| 10/08/2021 | OI030 | F | 300 | Palm Islands | -18.65251 | 146.59898 |
| 11/08/2021 | OI034 | F | 310 | Palm Islands | -18.65251 | 146.59898 |
| 11/08/2021 | OI036 | M | 197 | Palm Islands | -18.65251 | 146.59898 |
| 12/08/2021 | OI041 | F | 260 | Palm Islands | -18.65251 | 146.59898 |
| 18/09/2021 | OI056 | F | 300 | Palm Islands | -18.65251 | 146.59898 |
| 18/09/2021 | OI057 | F | 178 | Palm Islands | -18.65251 | 146.59898 |
| 19/09/2021 | OI059 | F | 280 | Palm Islands | -18.65251 | 146.59898 |
| 19/09/2021 | OI060 | M | 176 | Palm Islands | -18.65251 | 146.59898 |
| 6/11/2019 | 102T | F | 260 | Port Stephens | -32.80953 | 152.19961 |
| NA | TS204 | M | 400 | Port Stephens | -32.80953 | 152.19961 |
| NA | TS205 | M | 326 | Port Stephens | -32.80953 | 152.19961 |
| NA | TS209 | M | 375 | Port Stephens | -32.80953 | 152.19961 |
| NA | TS210 | F | 278 | Port Stephens | -32.80953 | 152.19961 |
| NA | TS211 | F | 378 | Port Stephens | -32.80953 | 152.19961 |
| NA | TS213 | M | 380 | Port Stephens | -32.80953 | 152.19961 |
| NA | TS220 | F | 150 | Port Stephens | -32.80953 | 152.19961 |
| NA | TS220CD | F | 150 | Port Stephens | -32.80953 | 152.19961 |
| NA | TS264 | M | 269 | Port Stephens | -32.80953 | 152.19961 |
| NA | TS266 | F | 412 | Port Stephens | -32.80953 | 152.19961 |
| NA | TS267 | F | 313 | Port Stephens | -32.80953 | 152.19961 |
| NA | TS270 | F | 350 | Port Stephens | -32.80953 | 152.19961 |
| NA | TS278 | M | 275 | Port Stephens | -32.80953 | 152.19961 |
| NA | TS283 | F | 325 | Port Stephens | -32.80953 | 152.19961 |
| NA | TS287 | M | 365 | Port Stephens | -32.80953 | 152.19961 |
| NA | TS288 | M | 260 | Port Stephens | -32.80953 | 152.19961 |
| NA | TS295 | F | 299 | Port Stephens | -32.80953 | 152.19961 |
| NA | TS296 | M | 238 | Port Stephens | -32.80953 | 152.19961 |
| NA | TS301 | F | 358 | Port Stephens | -32.80953 | 152.19961 |
| NA | TS302 | M | 307 | Port Stephens | -32.80953 | 152.19961 |
| NA | TS318 | F | 389 | Port Stephens | -32.80953 | 152.19961 |
| NA | TS319 | F | 387 | Port Stephens | -32.80953 | 152.19961 |
| NA | TS320 | M | 342 | Port Stephens | -32.80953 | 152.19961 |
| NA | TS330 | M | 323 | Port Stephens | -32.80953 | 152.19961 |
| NA | TS339 | M | 265 | Port Stephens | -32.80953 | 152.19961 |
| NA | TS344 | M | 326 | Port Stephens | -32.80953 | 152.19961 |
| 3/12/2020 | FN1 | F | 264 | Raine Island | -11.588367 | 144.031367 |
| 4/12/2020 | FN2 | F | 246 | Raine Island | -11.588367 | 144.031367 |
| 5/12/2020 | FN4 | F | 310 | Raine Island | -11.588367 | 144.031367 |
| 6/12/2020 | FN5 | F | 316 | Raine Island | -11.588367 | 144.031367 |
| 7/12/2020 | FN6 | F | 296 | Raine Island | -11.588367 | 144.031367 |
| 8/12/2020 | FN7 | F | 320 | Raine Island | -11.588367 | 144.031367 |
| 9/12/2020 | FN8 | F | 272 | Raine Island | -11.588367 | 144.031367 |
| 10/12/2020 | FN9 | F | 260 | Raine Island | -11.588367 | 144.031367 |
| NA | TS001 | M | 180 | Sunshine Coast | -26.64017 | 153.20399 |
| NA | TS001CD | M | 180 | Sunshine Coast | -26.64017 | 153.20399 |
| NA | TS046 | F | 290 | Sunshine Coast | -26.64017 | 153.20399 |
| NA | TS046CD | F | 290 | Sunshine Coast | -26.64017 | 153.20399 |
| NA | TS185 | F | 113 | Sunshine Coast | -26.64017 | 153.20399 |
| NA | TS186 | F | 131 | Sunshine Coast | -26.64017 | 153.20399 |
| NA | TS187 | F | 335 | Sunshine Coast | -26.64017 | 153.20399 |
| NA | TS188 | F | 290 | Sunshine Coast | -26.64017 | 153.20399 |
| NA | TS189 | F | 224 | Sunshine Coast | -26.64017 | 153.20399 |
| NA | TS190 | F | 285 | Sunshine Coast | -26.64017 | 153.20399 |
| NA | TS191 | F | 141 | Sunshine Coast | -26.64017 | 153.20399 |
| NA | TS192 | F | 270 | Sunshine Coast | -26.64017 | 153.20399 |
| NA | TS193 | F | 170 | Sunshine Coast | -26.64017 | 153.20399 |
| NA | TS194 | F | 217 | Sunshine Coast | -26.64017 | 153.20399 |
| NA | TS195 | F | 194 | Sunshine Coast | -26.64017 | 153.20399 |
| NA | TS196 | F | 183 | Sunshine Coast | -26.64017 | 153.20399 |
| NA | TS197 | F | 138 | Sunshine Coast | -26.64017 | 153.20399 |
| NA | TS198 | F | 116 | Sunshine Coast | -26.64017 | 153.20399 |
| NA | TS199 | F | 133 | Sunshine Coast | -26.64017 | 153.20399 |
| NA | TS200 | F | 135 | Sunshine Coast | -26.64017 | 153.20399 |
| NA | TS201 | F | 153 | Sunshine Coast | -26.64017 | 153.20399 |
| NA | TS202 | F | 132 | Sunshine Coast | -26.64017 | 153.20399 |
| NA | TS203 | F | 140 | Sunshine Coast | -26.64017 | 153.20399 |
| 6/11/2019 | 103T | M | 300 | Sydney | -33.8438 | 151.322 |
| 19/11/2019 | 105T | F | 230 | Sydney | -33.8438 | 151.322 |
| 11/04/2019 | 73T | M | 240 | Sydney | -33.8438 | 151.322 |
| 9/05/2019 | 87T | F | 350 | Sydney | -33.8438 | 151.322 |
| NA | TS216 | M | NA | Sydney | -33.8438 | 151.322 |
| NA | TS217 | M | 349 | Sydney | -33.8438 | 151.322 |
| NA | TS218 | M | 359 | Sydney | -33.8438 | 151.322 |
| NA | TS219 | F | 180 | Sydney | -33.8438 | 151.322 |
| NA | TS588 | M | 370 | Sydney | -33.8438 | 151.322 |
| NA | TS589 | M | 341 | Sydney | -33.8438 | 151.322 |
| 1/03/2019 | 60T | F | 131 | Tathra | -36.7381 | 149.9972 |
| 7/03/2019 | 61T | F | 168 | Tathra | -36.7381 | 149.9972 |
| 27/03/2019 | 67T | F | 320 | Tathra | -36.7381 | 149.9972 |
| 30/03/2019 | 70T | F | 215 | Tathra | -36.7381 | 149.9972 |
| 6/04/2019 | 72T | F | 150 | Tathra | -36.7381 | 149.9972 |
| NA | TS224 | M | 143 | Tweed Heads | -28.15654 | 153.5787 |
| NA | TS231 | F | 163 | Tweed Heads | -28.15654 | 153.5787 |
| NA | TS232 | F | 167 | Tweed Heads | -28.15654 | 153.5787 |
| NA | TS238 | M | 157 | Tweed Heads | -28.15654 | 153.5787 |
| NA | TS239 | F | 158 | Tweed Heads | -28.15654 | 153.5787 |
| NA | TS240 | M | 148 | Tweed Heads | -28.15654 | 153.5787 |
| NA | TS255 | M | 117 | Tweed Heads | -28.15654 | 153.5787 |
| NA | TS256 | M | 165 | Tweed Heads | -28.15654 | 153.5787 |
| NA | TS257 | F | 364 | Tweed Heads | -28.15654 | 153.5787 |
| NA | TS258 | F | 260 | Tweed Heads | -28.15654 | 153.5787 |
| 1/12/2018 | CH1 | M | 230 | Whitsundays | -20.261744 | 148.936613 |
| 1/05/2021 | CH100 | F | 410 | Whitsundays | -20.261744 | 148.936613 |
| 2/05/2021 | CH101 | M | 293 | Whitsundays | -20.261744 | 148.936613 |
| 3/05/2021 | CH102 | F | 294 | Whitsundays | -20.261744 | 148.936613 |
| 4/05/2021 | CH103 | F | 355 | Whitsundays | -20.261744 | 148.936613 |
| 5/05/2021 | CH105 | F | 310 | Whitsundays | -20.261744 | 148.936613 |
| 6/05/2021 | CH116 | F | 255 | Whitsundays | -20.261744 | 148.936613 |
| 7/05/2021 | CH119 | F | 350 | Whitsundays | -20.261744 | 148.936613 |
| 8/05/2021 | CH122 | F | 350 | Whitsundays | -20.261744 | 148.936613 |
| 9/05/2021 | CH125 | F | 239 | Whitsundays | -20.261744 | 148.936613 |
| 10/05/2021 | CH138 | F | 287 | Whitsundays | -20.261744 | 148.936613 |
| 11/05/2021 | CH145 | M | 323 | Whitsundays | -20.261744 | 148.936613 |
| 12/05/2021 | CH147 | F | 338 | Whitsundays | -20.261744 | 148.936613 |
| 13/05/2021 | CH148 | F | 198 | Whitsundays | -20.261744 | 148.936613 |
| 27/09/2021 | CH159 | F | 344 | Whitsundays | -20.261744 | 148.936613 |
| NA | CH167 | NA | NA | Whitsundays | -20.261744 | 148.936613 |
| 28/09/2021 | CH170 | F | 178 | Whitsundays | -20.261744 | 148.936613 |
| 28/09/2021 | CH171 | F | 375 | Whitsundays | -20.261744 | 148.936613 |
| 28/09/2021 | CH172 | F | 283 | Whitsundays | -20.261744 | 148.936613 |
| 28/09/2021 | CH173 | F | 335 | Whitsundays | -20.261744 | 148.936613 |
| 29/09/2021 | CH177 | F | 350 | Whitsundays | -20.261744 | 148.936613 |
| 30/09/2021 | CH181 | F | 350 | Whitsundays | -20.261744 | 148.936613 |
| 1/12/2018 | CH2 | F | 242 | Whitsundays | -20.261744 | 148.936613 |
| 1/10/2021 | CH207 | M | 352 | Whitsundays | -20.261744 | 148.936613 |
| 1/09/2019 | CH23 | F | 300 | Whitsundays | -20.261744 | 148.936613 |
| 1/09/2019 | CH25 | F | 334 | Whitsundays | -20.261744 | 148.936613 |
| 1/09/2019 | CH26 | F | 370 | Whitsundays | -20.261744 | 148.936613 |
| 1/12/2019 | CH35 | M | 345 | Whitsundays | -20.261744 | 148.936613 |
| 10/06/2019 | CH4 | M | 264 | Whitsundays | -20.261744 | 148.936613 |
| 1/12/2019 | CH40 | F | 310 | Whitsundays | -20.261744 | 148.936613 |
| 1/12/2019 | CH43 | F | 231 | Whitsundays | -20.261744 | 148.936613 |
| 1/12/2019 | CH47 | F | 321 | Whitsundays | -20.261744 | 148.936613 |
| 10/06/2019 | CH5 | M | 316 | Whitsundays | -20.261744 | 148.936613 |
| 1/01/2020 | CH57 | F | 360 | Whitsundays | -20.261744 | 148.936613 |
| 11/06/2019 | CH6 | F | 386 | Whitsundays | -20.261744 | 148.936613 |
| 1/01/2020 | CH60 | M | NA | Whitsundays | -20.261744 | 148.936613 |
| 1/01/2020 | CH61 | M | NA | Whitsundays | -20.261744 | 148.936613 |
| 11/06/2019 | CH7 | M | 335 | Whitsundays | -20.261744 | 148.936613 |
| 1/10/2020 | CH78 | F | 307 | Whitsundays | -20.261744 | 148.936613 |
| 2/10/2020 | CH79 | M | 217 | Whitsundays | -20.261744 | 148.936613 |
| 3/10/2020 | CH80 | F | 350 | Whitsundays | -20.261744 | 148.936613 |
| 4/10/2020 | CH81 | F | 355 | Whitsundays | -20.261744 | 148.936613 |
| 5/10/2020 | CH84 | F | 340 | Whitsundays | -20.261744 | 148.936613 |
| 6/10/2020 | CH88 | M | 339 | Whitsundays | -20.261744 | 148.936613 |
| 7/10/2020 | CH91 | M | 340 | Whitsundays | -20.261744 | 148.936613 |
| 8/10/2020 | CH92 | F | 252 | Whitsundays | -20.261744 | 148.936613 |
| 9/10/2020 | CH94 | F | 382 | Whitsundays | -20.261744 | 148.936613 |
| 10/10/2020 | CH96 | F | 348 | Whitsundays | -20.261744 | 148.936613 |
| 1/05/2021 | CH98 | F | 285 | Whitsundays | -20.261744 | 148.936613 |

**Table S2.** Pairwise estimates of *F*_ST_ among tiger shark sampling locations. Values in bold represent significance with 95% confidence intervals not overlapping with zero.

|  | 1 | 2 | 3 | 4 | 5 | 6 | 7 | 8 | 9 | 10 | 11 | 12 | 13 | 14 | 15 | 16 | 17 | 18 | 19 | 20 |
| --- | --- | --- | --- | --- | --- | --- | --- | --- | --- | --- | --- | --- | --- | --- | --- | --- | --- | --- | --- | --- |
| 1. Ballina | * |  |  |  |  |  |  |  |  |  |  |  |  |  |  |  |  |  |  |  |
| 2. Bundaberg | **0.002** | * |  |  |  |  |  |  |  |  |  |  |  |  |  |  |  |  |  |  |
| 3. Cairns | 0.001 | 0.000 | * |  |  |  |  |  |  |  |  |  |  |  |  |  |  |  |  |  |
| 4. Capricorn Coast | **0.009** | **0.008** | 0.005 | * |  |  |  |  |  |  |  |  |  |  |  |  |  |  |  |  |
| 5. Coffs Harbour | 0.000 | **0.002** | 0.000 | 0.007 | * |  |  |  |  |  |  |  |  |  |  |  |  |  |  |  |
| 6. Evans Head | 0.000 | 0.001 | 0.001 | **0.005** | 0.000 | * |  |  |  |  |  |  |  |  |  |  |  |  |  |  |
| 7. Gold Coast | **0.017** | **0.011** | 0.012 | 0.010 | **0.013** | **0.012** | * |  |  |  |  |  |  |  |  |  |  |  |  |  |
| 8. Indo NT | 0.006 | 0.000 | 0.002 | 0.009 | 0.003 | 0.002 | 0.011 | * |  |  |  |  |  |  |  |  |  |  |  |  |
| 9. Kiama | 0.001 | 0.002 | 0.000 | 0.009 | 0.000 | 0.001 | **0.018** | 0.007 | * |  |  |  |  |  |  |  |  |  |  |  |
| 10. Laurieton | 0.004 | **0.004** | 0.002 | **0.007** | **0.003** | **0.002** | 0.009 | 0.001 | 0.001 | * |  |  |  |  |  |  |  |  |  |  |
| 11. Mackay | **0.003** | **0.002** | **0.002** | 0.009 | **0.003** | **0.002** | **0.015** | **0.006** | **0.004** | **0.007** | * |  |  |  |  |  |  |  |  |  |
| 12. New Caledonia | 0.000 | 0.001 | 0.000 | 0.008 | 0.000 | 0.000 | **0.019** | 0.001 | 0.001 | **0.006** | **0.002** | * |  |  |  |  |  |  |  |  |
| 13. Norfolk Island | 0.000 | 0.000 | 0.000 | 0.000 | 0.000 | 0.000 | 0.000 | 0.000 | 0.000 | 0.000 | 0.000 | 0.000 | * |  |  |  |  |  |  |  |
| 14. Palm Islands | 0.000 | 0.000 | 0.000 | 0.003 | 0.000 | 0.000 | **0.019** | 0.004 | 0.000 | 0.000 | **0.002** | 0.000 | 0.000 | * |  |  |  |  |  |  |
| 15. Port Stephens | **0.033** | **0.034** | 0.036 | 0.053 | 0.032 | 0.035 | **0.097** | 0.058 | 0.041 | **0.044** | **0.037** | 0.039 | **0.078** | 0.041 | * |  |  |  |  |  |
| 16. Raine Island | 0.000 | 0.000 | 0.000 | 0.007 | 0.000 | 0.000 | 0.015 | 0.006 | 0.002 | 0.002 | 0.004 | **0.004** | 0.000 | 0.000 | 0.045 | * |  |  |  |  |
| 17. Sunshine Coast | **0.006** | **0.004** | **0.002** | 0.004 | **0.004** | **0.003** | 0.000 | 0.000 | **0.003** | 0.002 | **0.007** | **0.003** | 0.000 | 0.000 | **0.035** | 0.000 | * |  |  |  |
| 18. Sydney | **0.002** | 0.000 | 0.001 | 0.007 | 0.000 | 0.000 | 0.016 | 0.000 | 0.004 | 0.000 | 0.001 | **0.001** | 0.000 | 0.001 | 0.050 | 0.002 | 0.000 | * |  |  |
| 19. Tathra | 0.001 | 0.000 | 0.000 | 0.005 | 0.000 | 0.000 | 0.013 | 0.005 | 0.002 | 0.002 | 0.003 | 0.000 | 0.000 | 0.000 | 0.041 | 0.000 | 0.000 | 0.000 | * |  |
| 20. Tweed Heads | 0.002 | 0.001 | 0.001 | 0.006 | 0.002 | 0.002 | 0.016 | 0.004 | 0.006 | 0.003 | **0.006** | **0.002** | 0.000 | 0.003 | 0.043 | 0.004 | **0.002** | 0.000 | 0.004 | * |
| 21. Whitsundays | 0.001 | **0.001** | **0.002** | 0.004 | 0.001 | 0.000 | **0.013** | 0.004 | 0.003 | **0.001** | **0.002** | 0.002 | 0.000 | 0.002 | 0.036 | 0.002 | **0.002** | 0.000 | 0.000 | 0.000 |

**Table S3.** Relatedness categories (first-, second- and third-degree relationships) inferred from co-ancestry coefficients (θ) and accompanying R_0_ and R_1_ coefficients for kinship analyses using the package SNPRelate.

| Relationship | Degree Of Relatedness | K_2_ | K_1_ | K_0_ | θ= K_1_/4+ K_2_/2 |
| --- | --- | --- | --- | --- | --- |
| Clones |  | 1 | 0 | 0 | 0.5 |
| Full Siblings | 1^st^ | 0.25 | 0.5 | 0.25 | 0.25 |
| Parent Offspring | 1^st^ | 0 | 1 | 0 | 0.25 |
| Double First Cousin | 2^nd^ | 0.0625 | 0.375 | 0.5625 | 0.125 |
| Half Siblings, Grandparent/Grandchild, Aunt/Uncle | 2^nd^ | 0 | 0.5 | 0.5 | 0.125 |
| First Cousins | 3^rd^ | 0 | 0.25 | 0.75 | 0.0625 |
| Unrelated |  | 0 | 0 | 1 | 0 |


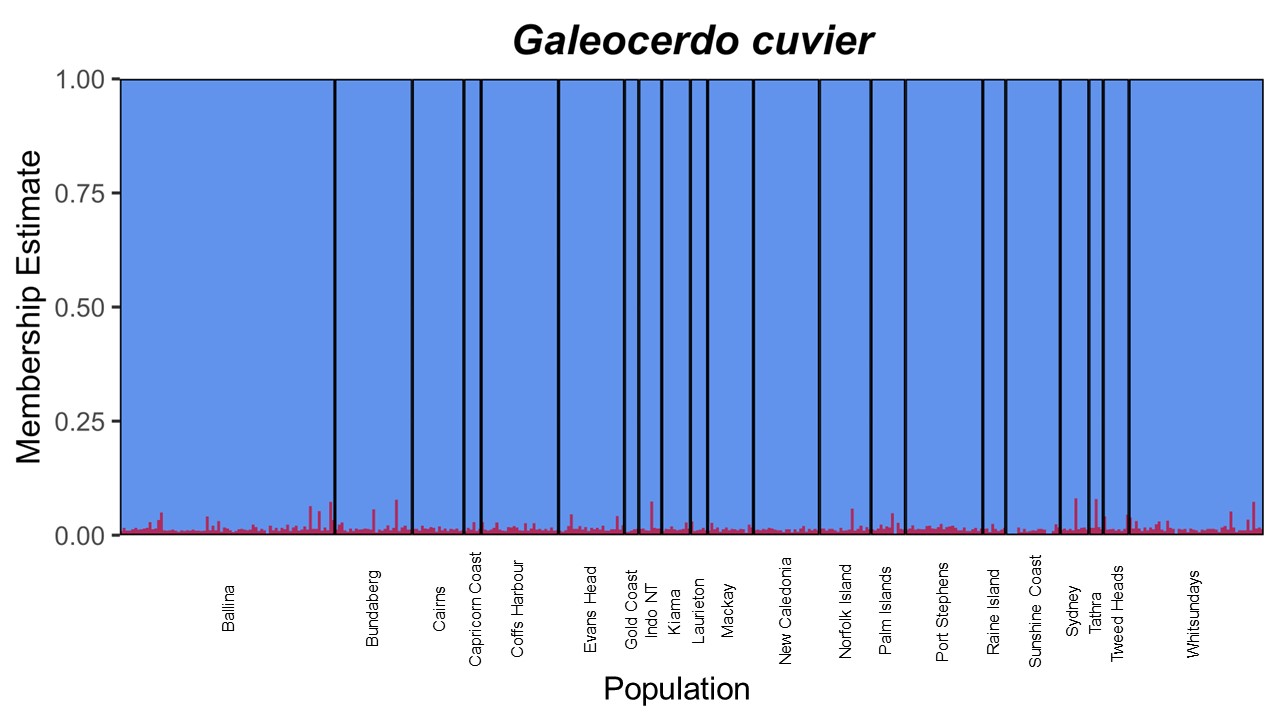


**Figure S1.** Bayesian STRUCTURE plot indicating a lack of genetic structuring (K=1). Single vertical lines represent individuals arranged into sites from which they were sampled, where coloured segments are proportional to the membership coefficient for each population cluster. No genetic differentiation between region is observed here.

**
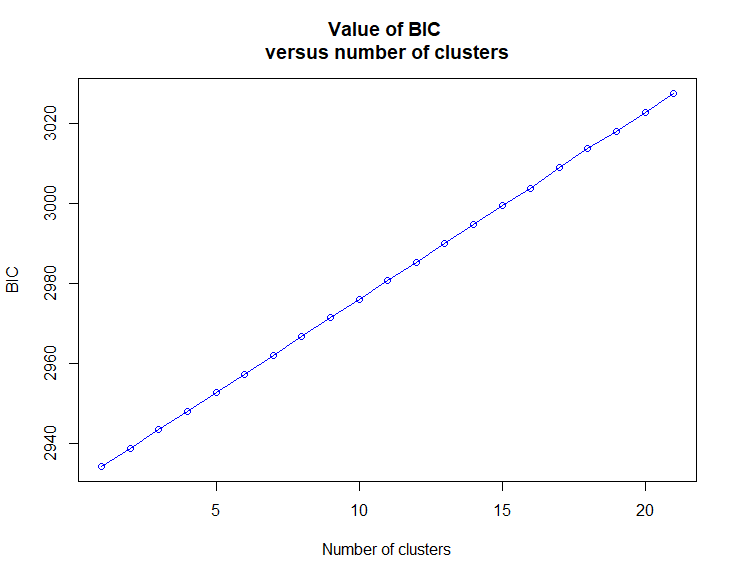
**

**Figure S2.** BIC plot from Discriminant Analysis of Principal Components (DAPC), indicating the most probably number of clusters to be 1.

**Figure S3.** Spatial autocorrelation results with coefficient (r) for SNP data over a range of geographic distance classes spanning 700 km for all tiger sharks.

**Figure S4.** Spatial autocorrelation results with coefficient (r) for SNP data over a range of geographic distance classes spanning 600 km for 50 (top) and 100 (bottom) randomly sampled female juvenile-subadult tiger sharks.
